# Automatic Tumor-Stroma Separation in Fluorescence TMAs Enables the Quantitative High-Throughput Analysis of Multiple Cancer Biomarkers

**DOI:** 10.1371/journal.pone.0028048

**Published:** 2011-12-02

**Authors:** Bernd Lahrmann, Niels Halama, Hans-Peter Sinn, Peter Schirmacher, Dirk Jaeger, Niels Grabe

**Affiliations:** 1 Institute of Medical Biometry and Informatics, University Hospital Heidelberg, Heidelberg, Germany; 2 Hamamatsu Tissue Imaging and Analysis Center (TIGA), BIOQUANT, Heidelberg, Germany; 3 National Center for Tumor Diseases, University Hospital Heidelberg, Heidelberg, Germany; 4 Department of Pathology, University Hospital Heidelberg, Heidelberg, Germany; Institute of Cancerology Gustave Roussy, France

## Abstract

The upcoming quantification and automation in biomarker based histological tumor evaluation will require computational methods capable of automatically identifying tumor areas and differentiating them from the stroma. As no single generally applicable tumor biomarker is available, pathology routinely uses morphological criteria as a spatial reference system. We here present and evaluate a method capable of performing the classification in immunofluorescence histological slides solely using a DAPI background stain. Due to the restriction to a single color channel this is inherently challenging. We formed cell graphs based on the topological distribution of the tissue cell nuclei and extracted the corresponding graph features. By using topological, morphological and intensity based features we could systematically quantify and compare the discrimination capability individual features contribute to the overall algorithm. We here show that when classifying fluorescence tissue slides in the DAPI channel, morphological and intensity based features clearly outpace topological ones which have been used exclusively in related previous approaches. We assembled the 15 best features to train a support vector machine based on Keratin stained tumor areas. On a test set of TMAs with 210 cores of triple negative breast cancers our classifier was able to distinguish between tumor and stroma tissue with a total overall accuracy of 88%. Our method yields first results on the discrimination capability of features groups which is essential for an automated tumor diagnostics. Also, it provides an objective spatial reference system for the multiplex analysis of biomarkers in fluorescence immunohistochemistry.

## Introduction

Automation in immunohistological image processing is currently an essential technological development taking place in the clinical hunt for objective biomarkers in research and diagnostics. In cancer research one of the most important but also extreme challenges is the development of methods for the automatic separation of tumor and stroma tissue [1], [2]. Success here will have an enormous impact on the applicability of biomarkers in routine cancer diagnostics and therapy as well the large-scale generation of histological tissue data for research purposes. An important method routinely used in this context which we here use to illustrate the problem is the Tissue Microarray (TMA) technology, introduced in 1998 [3]. TMAs allow the simultaneous immunohistochemical analysis of several hundred tissues on a single slide [4]–[6]. But as generally in all fields of pathology, the manual visual scoring of TMAs is routinely based on the quantitative analysis of protein levels by pathologists or other experts is subjective, labor-intensive, is time consuming and most importantly suffers from intra and inter-observer variability [7]. As a solution, fluorescent capable microscopic whole-slide scanners have become available recently but are still only rarely used although they will have a key role in transforming histological evaluation into objectivity. Fluorescence based staining here is essential as it overcomes the key problem of brightfield stains by the objective and automatic capturing of distinct biomarker signals [8]. Although fluorescence helps in the quantification of individual cells, it does not per se help in differentiating tumor and stroma. In fluorescence tissue slides are frequently counterstained with DAPI (4′,6-diamidino-2-phenylindole) taking the role of a conventional background stain. This makes the tumor-stroma separation more complex as the primary visual information of the tissue structure is much harder to recognize in the DAPI channel than in chromogenic histology. A histological biomarker which would exclusively stain tumor tissue is not available. Instead heterogeneity of spatial protein expression patterns is inherent to cancer. An excellent example here are the aggressive triple negative breast cancer tissues which do not express the genes for the most valuable prognostic marker like the estrogen receptor (ER), the progesterone marker (PR) and the human epidermal growth factor receptor type 2 (Her2) [9]. The absence of the expression patterns of these biomarkers disallows using any single one of them as a reference protein biomarker and renders it essential to separate the cancerous from the healthy/connective tissue by the help of objective, standardized processing algorithms based on morphological criteria. Thus, pathological evaluation routinely uses morphological criteria as a spatial reference system to determine the tumor area in cancer histology. We conclude that combining the advantages of fluorescence with automatic image acquisition and processing requires the development of algorithms for tumor-stroma separation solely from a DAPI background stain being frequently used in immunofluorescence.

**Figure 1:**
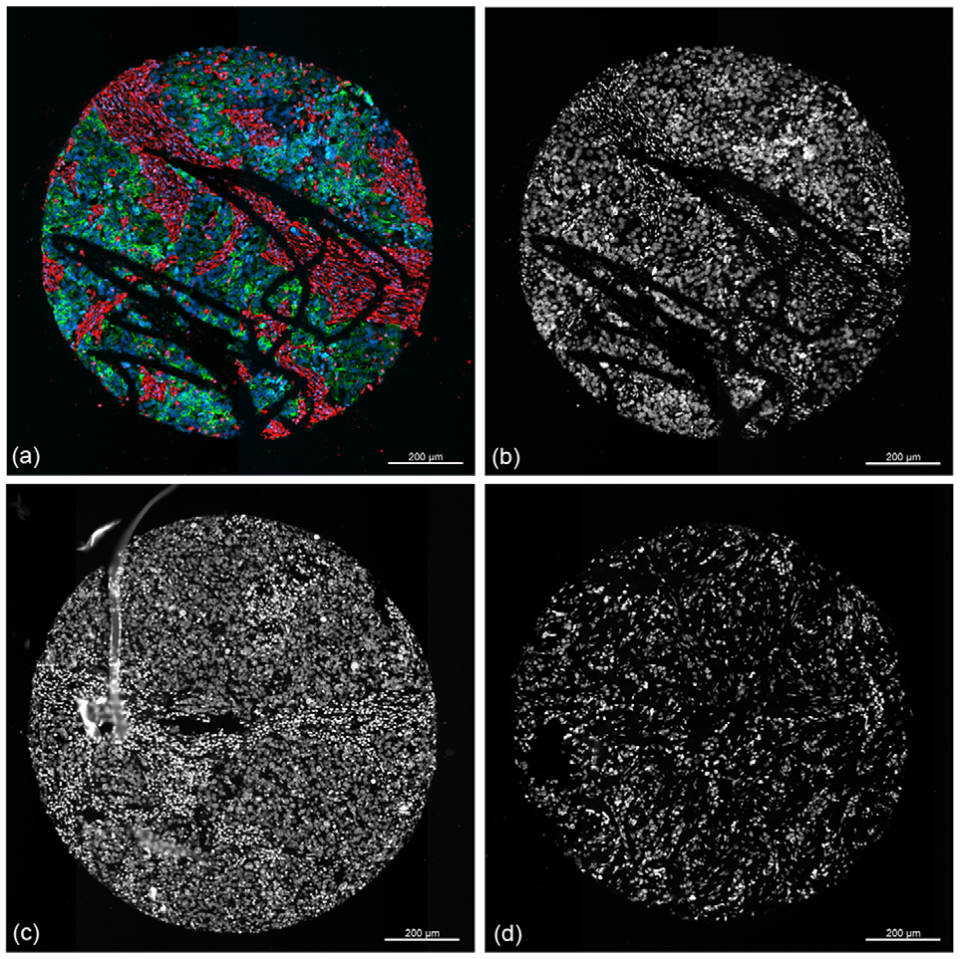
Microscopic image examples of different TMA-cores. (**a**) Representation of all 3 channel of a fluorescently stained core in RGB colorspace. Glyphs originated due to TMA's preparation. Red representing the stromal marker (Vimentin), green the tumor marker (CK19) and blue the DAPI channel highlighting the cell nuclei; (**b**) the DAPI channel of (**a**) as an intensity image: in general tumor cells are darker and tighter connected than stromal cells; (**c**) another DAPI image of a core with a high density of cells; (d) an example of a core with a lower density of cells **shows** the high heterogeneity among the cores.

Therefore, we here set out to develop such an automatic algorithm based only on the DAPI channel (Figure 1B–D). Several methods for the separation of cancerous tissue from other tissue types by morphological criteria are available in the literature. Amaral et al. [10], [11] present two different methods whereby color features are used for the classification of whole TMA-cores. In [12] textural features help to separate different tissue regions on a TMA and in [13] textural features are used for the detection of pathologic regions in histological slides. But all these methods work on chromogenic stained tissue samples where for the classification of the different tissue types the information of all 3 RGB channels was obtainable. Classifying tumor tissue only in the DAPI channel forces us to deal with less information available for the classification step compared to the previous other approaches. Only few publications deal with the classification of fluorescently stained tissues. In [14] the authors use nuclear features obtained from the DAPI-channel to distinguish whether the whole tissue is cancerous or healthy instead of classifying the different types present on the tissue. Most of the published work in biomarker research use two biomarkers for co-localization or manually segment the cancerous tissue instead of an automated manner [15]–[18].

Gunduz et al. [19] published a novel method for the classification of chromogenic stained brain tissue samples. They formed cell graphs based on the topological distribution of the tissue cells and extracted the corresponding graph metrics to train a classifier. The classifier was able to distinguish between cancerous and healthy tissue. A graph here is an abstract representation of objects (nodes) where pairs of these objects are linked by edges. The method was further developed in [20] and [21]. Bilgin et al. [22]–[23] demonstrated that they successfully analyzed breast and bone tissue samples by the help of cell graphs. They evaluated their method on hand-selected and non-biomarker characterized breast cancer samples.

Here we further developed this approach by developing a novel method capable of classifying fluorescently stained Tissue Microarrays. Our method uses cell graphs based on three different categories of features reflecting the properties of the cells contained in the graph (nodes) and their similarity (edges). From a potential set of features we determine those which are best capable of separating tumor and stroma tissue. Clearly, performing an accurate tumor-stroma separation is already a challenging task. Using furthermore only the DAPI-channel for this task requires an even higher performance in segmentation and classification.

As the first step we performed watershed segmentation and then we built cell graphs by linking the segmented cell nuclei under each other. The linking of the cells is based on new rules especially adapted for fluorescently stained TMAs which can consist of several different tissues types. Instead of using only topological graph metrics for the cell-graph classification, we also determine the morphological and intensity based cell features of each cell-graph. By combining all three feature types we were able to obtain a successful tissue classifier for fluorescent histological slides.

We demonstrate our method on 180 core images of TMAs from invasive triple negative breast cancer biopsies containing cancerous tissue as well as stroma (connective tissue). Our method method was able to separate tumor and connective tissue that coexist on the same tissue core with a total overall accuracy of 88.80(±07.73) %.

## Materials and Methods

### Tissue samples

The data set total consists of 210 tissue microarray core images of invasive triple negative breast cancer biopsies obtained from 6 TMAs. The tissue was obtained from the tissue bank of the National Center for Tumor Diseases (NCT) at the University Hospital Heidelberg. Obtaining tissue samples was approved by the ethics committee of the Medical Faculty Heidelberg. According to the official regulations of the University's Tissue Bank determined by said ethics committee no individual consent has to be obtained from individual patients for patient samples older than 3 years. Documentation of all procedures are handled in an ISO certified process by the NCT tissue bank. Each TMA contains two cores of 1 mm diameter from 42 different patients (total 84 cores per TMA). One core is obtained from the periphery of the tumor and the other is obtained from the tumor's center. We excluded cores from our data set if their area was below fifty percent of a regular core or if unusable. Each image is taken in a 20 fold magnification and has an average size of 2800×2900 pixels. All TMAs are stained with 3 fluorescent dyes. Every TMA was stained with DAPI highlighting the cell nuclei as a counterstain The other used antibodies (Vimentin, CK19 and CK5/6) were conjugated with Alexa Fluor® 488 (FITC alternative, green fluorescent dye) or Alexa Fluor® 594 (red dye). Figure 1A illustrates a tissue core stained with 2 different biomarkers and DAPI as counterstain. Figure 1B–D illustrates further representative examples of the DAPI channel of three different tissue-cores.

### Image acquisition

Fluorescently stained TMAs were automatically imaged using the Nanozoomer HT Scan System (Hamamatsu Photonics, Hamamatsu Japan) capable of scanning whole slides. Glass slides were scanned at 20 fold magnification (resolution of 0.46 µm/pixel). For the scanning of the glass slides, the slide scanner automatically detects the region of interest that contains the array of cores and also determines automatically a valid focal plane for scanning. The resulting virtual slides had an averaged file size of 5 GB. Single core images with an average size of 2800×2900 pixels were located and extracted from the TMAs using template matching [24].

### General image analysis workflow

The key concept in this manuscript is the cell graph which we use to capture the topological cell distribution in tissues as well as the spatially related local cell features for classification. The major steps in this approach are the segmentation of the cell nuclei in the DAPI channel using watershed segmentation, the construction of the cell graphs, extracting the topological and local cell features from these graphs and use them to train a classifier. Image processing algorithms were developed using Matlab™ (Mathworks, Natick, Mass., USA) with the image processing toolbox.

**Figure 2:**
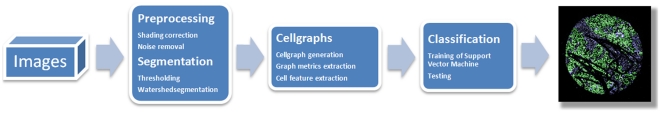
A Flowchart showing the single steps of our methodology. After obtaining the images, pre-processing steps enhance the image quality and watershed segmentation for the subsequent segmentation is applied. Accordingly the cell graphs are generated and features are computerized. The last step uses a SVM to classify the graphs as either tumor or stroma.

Our image analysis pipeline contains the following conceptional steps (as illustrated in Figure 2):

2.1 Pre-processing: We first applied several image enhancement methods to prepare the image for the subsequent segmentation step.

2.2 Cell segmentation: A Watershed-Transformation was applied for the cell nuclei segmentation.

2.3 Cell graph generation: Based on the segmented nuclei we generated cell graphs which represent the topological distribution of the nuclei on the tissue cores. We calculated several features for every (sub-) graph and also calculated intensity and morphological base features for every single nucleus on a core.

2.4 Classification and feature selection: A Support Vector Machine was trained for classification step and the F-Score was calculated for feature selection.

### 2.1 Preprocessing

**Figure 3:**
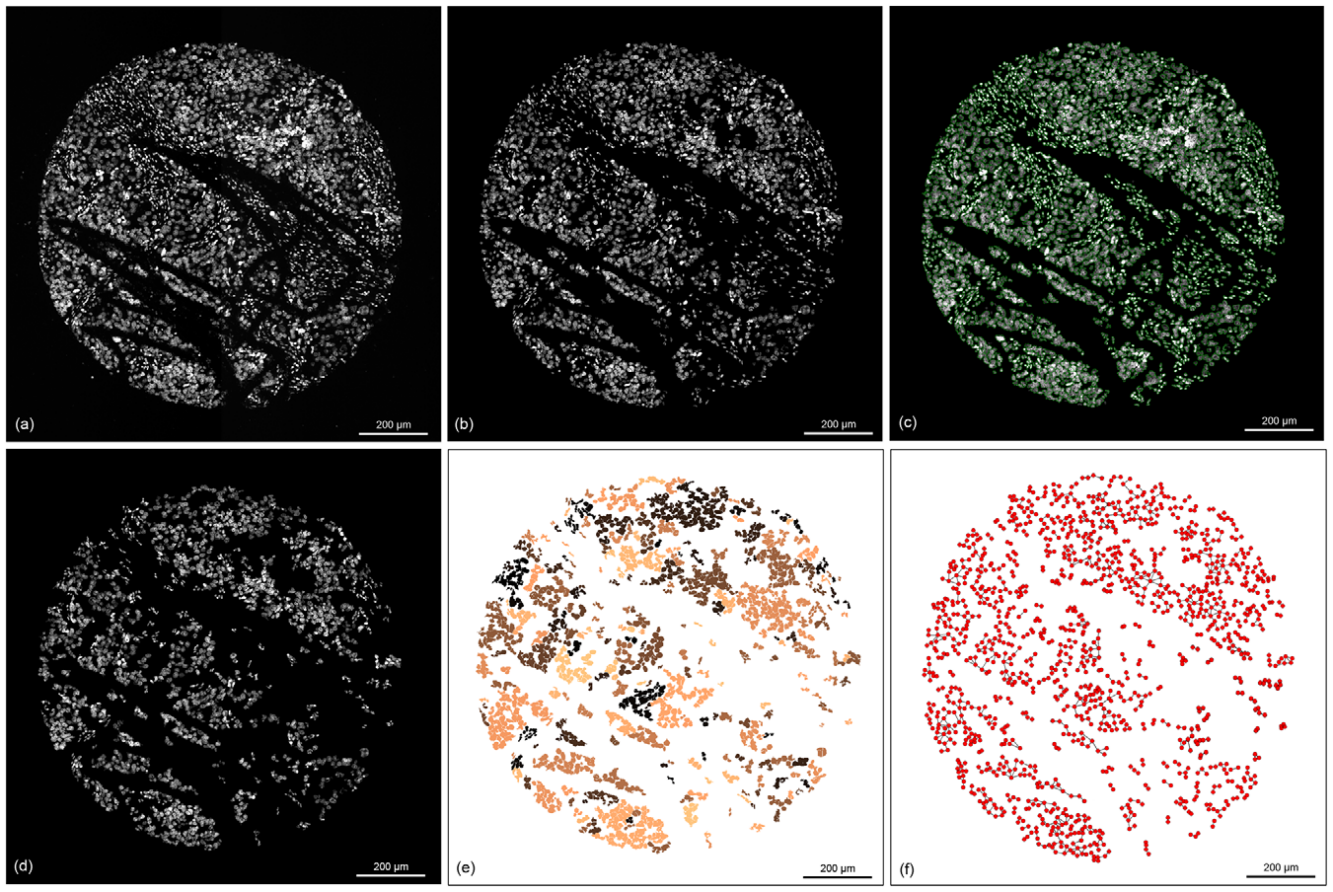
The different image processing steps and the graph generation steps. (**a**) original image of the DAPI-channel; (**b**) image after shading correction and noise removal; (**c**) result of the watershed segmentation, the segmented cells are highlighted by green contour; (**d**) the image after removal of single cells; (**e**) showing the cells which were connected via the graph generation step in the same color (cells marked with the same color belong to the same sub-graph); (**f**) cell graph representation of the cells. The red dots are the nodes which represent the cells, the black lines are the edges between them.

In this first step, we applied several methods to enhance the quality of the core image for the subsequent classification. We started to remove shading artefacts, which delineate the result of various optical phenomena such as lens vignetting or photo bleaching. Shading artefacts in fluorescence imaging can also be caused due to auto fluorescence of the samples or the mounting medium. Shading correction (flat field compensation) was used to compensate for lens vignetting as well as for inhomogeneity in the illumination. Shading correction was achieved by performing a black balance calibration using clear background areas. The next step in the image processing pipeline was the removal of noise and other small particles which were not suitable for later analysis. To exclude unspecific and diffuse background staining all pixels with intensity levels below a threshold of 25 were set to zero. A Median-Filter with a 3×3 kernel was used to smooth the image. The resulting image was converted into a binary image (by the use of Otsu's method [25]) in which objects with an area smaller than 150px (smaller than the size of connective tissue nucleus) are removed. Objects outside the regular core shape were removed using morphological operations like closing or opening combined with area filter. Eventually, isolated nuclei were observed inside the core. We assumed that these isolated nuclei belong to non tumerous cells and were thus excluded from the tumor tissue. To this end, we determined the smallest bounding box of the objects and expanded it by 20px in each direction. Based on this new coordinates, an image was cropped from the original binary image and the present objects in this image were counted. If only one object was present, the object was removed while the presence of more than one object implies contact to other cells and the object remained. Furthermore, in several cores we discovered large overstained areas with maximum intensity levels. These areas, which could be caused by agglomerated connective tissue cell nuclei at the TMA preparation or to high exposure times, are not suitable for further analysis and were removed. Figure 3B shows the results of the preprocessing step.

### 2.2 Cell Segmentation

Automated cell segmentation in fluorescently stained TMA can be problematic for reasons that include cell overlapping or clustered cells, complex tissue structure, debris and uneven background intensity due to auto fluorescence. Another difficulty is intensity variation between the nuclei which can lead to over-segmentation of the cell nuclei. Due to these intensity variations among nuclei, we first divided the image into one image representing only objects with a brighter illumination and one representing the darker objects. We then applied the segmentation step separately on both of these images. This separation was done by calculating a threshold based on Otsu's method [25] ignoring background pixels. A segmentation algorithm that has proven to be very useful for many nuclei or cell segmentation cases is the watershed segmentation [26]–[28]. We applied seeded watershed segmentation for the segmentation. Seeded watershed segmentation means, that starting regions, which are called seeds, are given as input to the watershed segmentation. We set the seeds in an automated way using the h-maxima transform [29]. The result of this segmentation step is shown in Figure 3C.

### 2.3 Cell graph Generation

**Figure 4:**
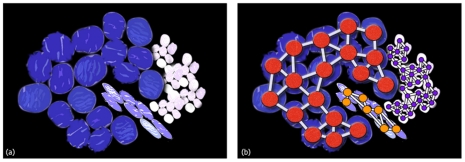
Conceptional representation of cell graphs. (**a**) Artificial sketch of 3 different 3 cell type: tumor cells in blue, lymphocytes in white and in purple fibroblast. (**b**) Cell graph representation of (a). Cells are depicted as nodes and links between them represent biological relations.

A graph is denoted as a set of objects where some pairs of objects are connected by links. The connected objects are represented by mathematical abstractions called nodes (also called vertices), and the links that connect some pairs of nodes are called edges. Formally, a graph is an ordered pair *G = (V,E)* where *V* is the set of nodes and *E* the set of edges linking the nodes of *V*. In our work, each of the former segmented cell nuclei was used as a node. Figure 4 shows a conceptional representation of cell graphs.

Different approaches are presented in literature for generating cells graphs, which represent the topological behaviour of tissues or cells in different scientific questions [19], [21]–[23], [30]. In [19] Gunduz et al. make use of the Waxman model for the cell graph generation. Bilgin et al. [22] and Gunduz et al. [21] use a probability function for linking the cells among themselves. In their approaches the probability of linking cells decays with a growing Euclidian distance between the cells centroids. In [23], [30] cells are simply linked if the Euclidian distance between their centroids is below a specific distance. Tumor cells generally appear in clusters, accordingly they can be expected in a marginal distance of another or appearingly “touching” each other. Thereby, this “touching” of nuclei occurs because of the three dimensional structure of the histological sections. By using the nuclei centroids for distance measurements alone it is possible that cells get linked although they are more apart than typical tumor cells. In our case, we are performing a pre-classification by only building links between cell nuclei touching each other and thereby excluding solitary cells (of connective tissue origin) from the graph construction step. In our method we test if cells touch each other by the following steps. We first convert the result of the watershed segmentation into a binary image and then we dilate each of the segmented cell nuclei separately. The dilation of an (cell nucleus)-Image *I* with a structuring element *S*, denoted as *I⊕S*, is defined as the set operation 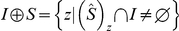 where Ŝ denotes the symmetric structuring element. We chose a diamond-shaped structuring element with a distance from the origin of 2. We then determine, if the cell nuclei were in very close contact (“touching” appearance) and set a link between them, if their pixel intersection was not an empty set after the dilation step:
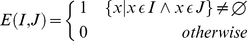
(1)where *I and J* are the particular images of two neighboring cell nuclei. In tissues, tumor cells are eventually tightly surrounded by connective tissue cells which could, after applying the above described distance rule lead to structural errors in the cell graph. Usually, the tumor cells are appearing with lower intensity levels than the connective tissue cells. Hence we link only cells, if the difference between their intensity levels is lower than a specific threshold. This threshold is dependent on variations in staining and fluorescence signal acquisition efficiency. We here empirically determined a difference of 30 intensity values as an applicable threshold for our data set. Concluding, neighboring cell nuclei with an intensity difference below this threshold are linked:
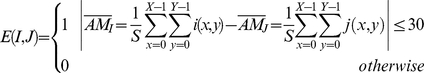
(2)Where 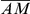 is the arithmetic mean of the cell image intensity level, X the number of rows, Y the number of columns and S = X*Y. Summing up, setting a link between two cell nuclei in our model depends on the probability of touching each other and that the difference of their intensity levels is lower than a specific threshold. Figure 4D shows an example image whereby single cells are removed. Figure 4E highlights the cell nuclei, which were linked through this graph generation step in the same color. A visual graph representation of this step is shown in Figure 4F. Cells which were not connected during the graph generation process were treated in an additional step described in section “single cell classification”.

### Cell Graph Features

After generating the cell graphs, we computed several features for the training of the classifier. In total we computed 22 features which can be divided into three different categories. The first 10 features, in literature usually called graph metrics [19], [23], capture the topological behavior of the graphs like the number of cells in a graph, the number of links between them or further topological relations among the cells (feature category T). The remaining 12 features capture morphological properties (feature category M) like area, shape as well as intensity based properties (feature category I) of the single cell nuclei of a graph and were chosen based on their expected suitability. The last two categories of features are first computed for each single cell nucleus and then the average is used as a feature of the corresponding graph. Keep in mind that several of these intensity based features depend on the imaging conditions like the exposure time, the concentration of the biomarker, time lag between staining and imaging due to photo bleaching and further more. It requires that those conditions remain constant across the datasets. In Table 1 the applied features and graph metrics are described in detail.

**Table 1:** Graph metrics used to train the classifier and their description.

Name	Description
**Graph metrics (topological features)**	
(1) Nr. of nodes	Defines the number of nodes in a graph.
(2) Nr. of edges	Total number of edges in a graph.
(3) Average degree	The average degree of the nodes of the graph. The degree of a node is defined as the number of its edges. It explains the number of neighbour nodes.
(4) Diameter	The eccentricity *ε(u)* of a graph node *u* in a connected graph *G* is the maximum graph distance between *u* and any other node *v* of *G*: 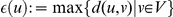 The maximum eccentricity is the graph diameter.
(5) Radius	The minimum graph eccentricity is called the graph radius: 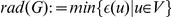
(6) Nr. of central points	Number of nodes that have eccentricity equal to the radius.
(7) Average clustering coefficient	Here, the average clustering coefficients of the nodes of the graphs are used as a global metric. The clustering coefficient C_i_ of a node v_i_ is given as: 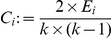 Where *k* is the number of neighbours of node *i* and *E_i_* is the number of existing edges between its neighbours.
(8) Nr. of end nodes.	The number of nodes with degree equal one.
(9) Percentage of end nodes.	The percentage of end nodes of a graph.
(10) Hop-plot exponent	The hop-plot exponent is computed by the slope of the hop-plot values as a function of h in log-log-scale. The hop-plot value reflects the size of a neighbourhood between nodes within a hop *h*. For hop *h*, the hop plot value is defined as the number of node pairs such that the path length between these pairs is less than or equal to h hops.
**Averaged node features (morphological and intensity features)**	
(11) Average area	The average area of the cells of the graph.
(12) Average eccentricity	The eccentricity is the ratio of the distance between the centroid of the ellipse and its major axis length. The value is between 0 and 1 (an ellipse whose eccentricity is 0 is actually a circle, while an ellipse whose eccentricity is 1 is a line segment.). Eccentricity is given as: 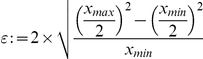 Where *x_max_* is the major axis length and *x_min_* the minor axis length.
(13) Average equivalent diameter	The equivalent diameter specifies the diameter of a circle with the same area as the cell: 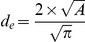 Where *A* is the area of the cell.
(14) Average extent	The extent specifies the proportion of pixels in the smallest rectangle containing the cell that are also in the region. Computed as the cell area divided by the area of the smallest rectangle containing the cell.
(15) Average major axis length	Major axis length specifying the length of the major axis of the ellipse that has the same normalized second central moments as the cell.
(16) Average minor axis length	Minor axis length the length (in pixels) of the minor axis of the ellipse that has the same normalized second central moments as the cell.
(17) Average max Intensity	Maximum intensity of the cell.
(18) Average min Intensity	Minimum intensity of the cell.
(19) Average mean intensity	Mean intensity of the cell.
(20) Average perimeter	Perimeter: the distance around the boundary of the cell.
(21) Average STD Intensity	Standard deviation of the intensity of a cell.
(22) Average median intensity	Median intensity level of a cell.

### 2.4 Classification and feature selection

Support vector machines (SVMs) [31] are commonly used as supervised learning methods for classification in computational biology and image processing tasks [32]–[34]. Starting point for the training of a SVM is a set of training data whose class membership is known:
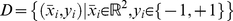
(3)where 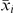 are the feature vectors and 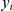 their respective class labels (tumor cells or connective tissue cells). The SVM maps these input vectors into a higher dimensional space and constructs an optimal hyper plane separating the data into two groups. By solving a quadratic programming optimization problem, the SVM calculates the normal vector 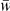 and the bias b of the separating hyper plane 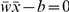 which maximizes the margin between the support vectors 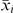 of different classes. The width of the margin is equal to 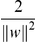, thus the widest margin between the vectors is found by minimizing under the restrictions 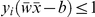, requiring a separable data set. The hyper plane then is used as a sign function for the classification of each feature vector of the test set. The classification function returns either +1 if the test data is member of the class, or −1 if it is not. When perfect separation is not possible, a slack variable 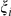 is introduced for each vector 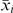. The constraints for computing the optimal hyperplane are then formulated as 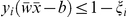 and the hyperplane can be found by minimizing:
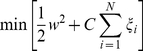
(4)where *C* is a cost parameter that determines the effect of outliners on the resulting hyper plane. The described SVM is capable to separate linear data. To create a classifier which is able to classify nonlinear data the kernel trick is applied. The key idea is to transform 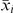 into a higher dimensional space to find a separating hyper plane using a kernel. This allows the algorithm to fit the maximum-margin hyper plane in a transformed feature space. Equation 4 can be rewritten as (5):
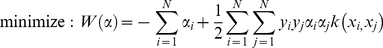
(5)

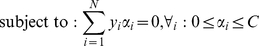
(6)where the 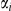 values are the Lagrange multipliers, which can be positive or negative, due to the equality constraints and 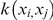 is the kernel function. In this article, we used a radial basis kernel (RBF) 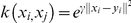 which is also known as Gaussian kernel.

### Feature Selection

We calculated the F-score for the selection of the features included in the SVM. Feature selection is a technique to find a subset of features by removing most unimportant and redundant features from the feature space. This technique generally helps to improve the total performance of the classifier, speeding up the learning process, allows a better representation of important features and results in a remaining feature set with maintained discriminatory power. The F-score measures the discrimination between two sets of features [35]. A higher F-score indicates to a higher discriminate feature than a feature with a lower F-score. We calculated the F-score for each feature *i* as described in (7) with the given training vectors 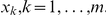:
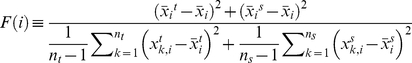
(7)where 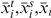 are the mean values of the *i*th feature of the tumor, stroma, and whole data set. 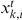 is denoted as the *i*th feature of the tumor instance and 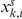 the *i*th feature of the stroma instance.

### Single cell classification

Based on the two criteria for the cell graph generation (intensity and distance), it can occur that individual cells are not linked to any other cell. Thus, these cells are not included in the classification step and we treat them with an additional algorithm in a separate single cell classification step. We first try to identify inflammatory cells (lymphocytes e.g.) and fibroblasts which are part of the stromal class. Usually, inflammatory cell nuclei appear as small roundish cell nuclei with a very high intensity compared to other cells on the core. Cell nuclei are therefore classified as inflammatory cells when: the cell nucleus intensity is higher than a specific level, a metric which calculates the roundness is higher than a threshold and the area is smaller than 500 pixels:
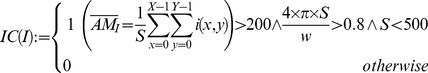
(8)where 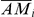 is the arithmetic mean intensity, S = X*Y the area and w the perimeter of a cell nucleus. Fibroblasts generally have an elliptical shape and were identified by:
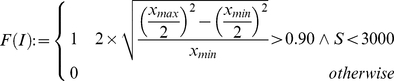
(9)where 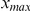 is the major- and 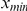 the minor-axis of the cell nuclei. These values are utilized to calculate the eccentricity of an ellipse. The eccentricity of a circle is 0 and an ellipse which eccentricity is 1 is a line segment. The remaining cell nuclei were classified by the use of a support vector machine. We used the 12 morphological and intensity based features already mentioned in section “Cell Graph features” to classify each single cell nucleus. We trained the SVM with the single cell nuclei of our training set and evaluated the algorithm separately as depicted in result section.

## Results

**Figure 5:**
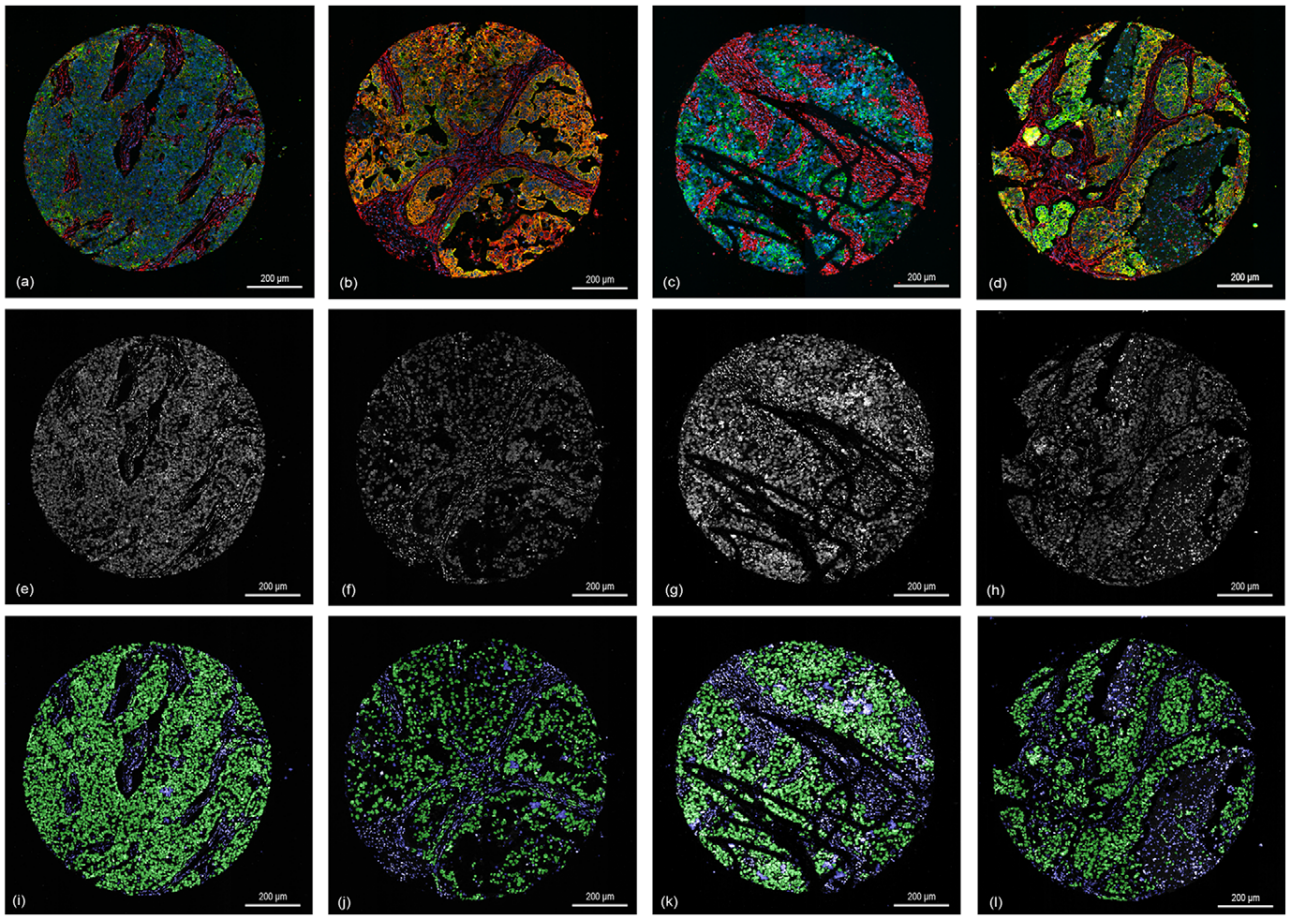
The results of the classification. (a–d) showing the original RGB core images; (e–h) showing the corresponding DAPI channel as an intensity image of the cores (a–d); (i–l) results of the classification step, green = cells classified as tumor cells, blue = cells classified as stroma cells.

The overall goal of our approach was to automatically classify each cell of a TMA core by the help of the generated cell graphs. The training and the classification is based only on the DAPI channel primarily staining the nuclei. Figure 5 illustrates the results of our approach on 4 different TMA-cores.

### Cell segmentation step

The cell nuclei segmentation was evaluated on 3 randomly selected real core images obtained from one TMA. In total 5162 nuclei were used and ground truth was obtained from one expert who marked the over- and under-segmented cell nuclei. The watershed algorithm proposed here can correctly segment 94.1%(±3.75) of the nuclei. Table 2 shows the detailed segmentation results and Figure 3C shows an example of this step whereby the segmented nuclei are surrounded by a green contour.

**Table 2:** Accuracy of the watershed cell segmentation.

	Total Nuclei	Correctly segmented	Over-segmented	Under-segmented
**Sum**	5162	4860	272	30
**Percentage**	100%	94.1(±3.75)	5.3(±4.0)	0.6(±0.3)

### Feature selection

Feature selection simplifies and shortens training of a classifier, and frequently also improves its accuracy. For feature selection from 30 core image we first generated in total 7888 topologically disjunct cell graphs leading to use the same total number of feature vectors. This total set of features comprises 4065 feature vectors for the tumor class and 3823 for the stroma class. The feature values occur within largely varying numerical ranges. Therefore we normalized them to the range of [0,1] to enhance the learning progress.

We calculated the F-score (discriminative power of a feature) for each of the 22 features from Table 1 to determine the best feature set for the classification task. Based on the results shown in Table 3 we picked the best 15 features for the training of the support vector machine. Our results allow the comparison the discriminative power of the three studied categories (T, I, M) of features. We find that the most predictive features are the morphological and intensity based features like the standard deviation of the intensity of a cell graph, the minor axis length of the cell nuclei within a cell graph and the equivalent diameter of them. The features with the lowest performance belong to the graph metrics: the number of central points, the number of end nodes and the number of edges.

**Table 3:** The F-scores of each feature in descending order.

Features	Type	F-score
**Average STD Intensity**	I	0.240
**Average minor axis length**	M	0.212
**Average equivalent diameter**	M	0.209
**Average area**	M	0.182
**Average max intensity**	I	0.143
**Average mean intensity**	I	0.108
**Average perimeter**	M	0.063
**Average median intensity**	I	0.056
**Average major axis length**	M	0.051
**Hop-plot exponent**	T	0.039
**Average degree**	T	0.038
**Percentage of end nodes**	T	0.038
**Average eccentricity**	T	0.038
**Diameter**	T	0.038
**Radius**	T	0.038
Average extent	M	0.038
Average min Intensity	I	0.016
Nr. of nodes	T	0.008
Average clustering coefficient	T	0.007
Nr. of edges	T	0.006
Number of end nodes	T	0.004
Number of central points	T	0.001
**Average morphological features**	M	0.144
**Average intensity features**	I	0.112
**Average topological features**	T	0.023

The table shows the evaluated features sorted by their decreasing value for tissue classification (F-score). For each feature it is given whether it is of morphological (M), intensity (I), and topological character (T).

### Cell-graph Classification

For the training of the SVM we used a training set of 30 cores obtained from one slide and with the above described feature set. The remaining 180 cores were used as test set and are obtained from 5 other slides. Images used in the test set were excluded from the training set. For training the classifier we manually segmented the 30 cores into tumor and stroma according to the used biomarkers. Vimentin was used as stromal marker. Although it is partly expressed in tumor cells Keratin 5/6 and 19 allowed the distinct segmentation of the tumor. Cores where no stain for the keratins was observed were excluded from the study. We determined the total areas of both classes in the data set averaged over all cores, the area of tumor is 64.6% whereas the area of stroma is 35.4%. Table 4 shows the averaged accuracies of the training set, the 5 slides of the test set and the total averaged values of the test set. We present the overall accuracies and the producer's accuracy of the tumor and the stroma class. Averaged over the test set, we achieved a total overall accuracy of 88.80(±07.73). For the prediction of the tumor class we achieved an average accuracy of 88.02(±13.51) and for the stroma class an accuracy of 84.67(±11.80).

**Table 4:** The average classification accuracies.

	Training set	Test set					Average 1–5
		1	2	3	4	5	
Overall	88.47(±06.68)	87.65(±08.19)	90.30(±06.44)	88.68(±07.19)	88.76(±06.98)	88.59(±09.83)	88.80(±07.73)
Tumor	89.26(±10.20)	87.56(±13.29)	87.83(±12.47)	88.00(±17.64)	88.98(±10.01)	87.71(±14.13)	88.02(±13.51)
Stroma	85.14(±10.95)	81.19(±11.62)	91.45(±06.21)	82.97(±15.12)	80.02(±12.35)	86.90(±13.69)	84.67(±11.80)

The table shows the accuracies of the training set and the accuracies of the slides from the test set.

As mentioned in the method section, some of the cell nuclei were not linked during the cell-graph generation step with a cell-graph because they are too far from other cells or the difference between their intensities does not allow linking them. We added an optional algorithm for the classification of these cells and present the results in Table 5. By the use of this single cell classification algorithm we achieved an overall accuracy of 86.12(±07.46), an accuracy of 86.60(±13.22) for the tumor class and 79.75(±13.27) for the stroma class. The use of the single cell classification therefore reduces the total accuracy but gives the advantage to classify each cell available on the core.

**Table 5:** The accuracies of the slides of the test set with the additional single node classification.

	Test set					Average 1–5
	1	2	3	4	5	
Overall	84.02(±07.83)	87.46(±6.85)	86.12(±08.23)	86.28(±05.88)	86.69(±08.51)	86.12(±07.46)
Tumor	85.62(±13.26)	86.05(±12.57)	86.31(±17.08)	88.39(±09.77)	86.64(±13.41)	86.60(±13.22)
Stroma	75.94(±13.11)	84.58(±13.63)	80.23(±14.24)	76.29(±11.81)	81.72(±13.55)	79.75(±13.27)

## Discussion

The high throughput analysis of fluorescently stained histological slides is becoming of increasing importance. To biomedical studies it offers the great advantage of objective biomarker quantification in individual color channels. Unfortunately, it in the same time results in an increased difficulty of distinguishing tumor and stroma tissue. We therefore here presented a new method capable of classifying fluorescently stained histological slides into tumor and stroma in the DAPI channel. The restriction to the DAPI channel severely reduces the available information for the image processing steps compared to the other methods based on all RGB color channels. We demonstrate that our approach is capable of separating the tumor from other tissue kinds on the same core in triple negative breast cancer as an example of higly heterogeneously expressing tumor tissue.

Technically, we make use cell graphs which have been used previously deployed for chromogenic tissue. As we did not know which kind of features might be effective for classifying fluorescence tissue we combined different kinds of features: topological features (T), morphological (M) and intensity based features (I) of the single cell nuclei related to the particular cell graph. We performed a systematic and quantitative evaluation of the value the individual features contribute to the discrimination between tumor and stroma tissue. Our results showed that the most relevant features indeed do not belong to the previously in literature used topological metrics but belong to the class of either morphological or intensity based features. The integration of morphological and intensity based features into the classification step significantly contributed to the increase in the classifiers power to discriminate tumor and stroma. The use of a broad spectrum of feature types reflects the complex visual tasks pathology perform in their daily histological assessments.

For segmentation we used the watershed algorithm. Nuclei were linked if they were close to each other and the difference of their intensity level is in a certain threshold. The linked cell nuclei then formed the cell graphs representing the topological structure of the whole tissue core. After segmentation the support vector machine was trained using the extracted features. Our evaluation on a total test set of 180 single core images yielded an overall accuracy of 88.80(±07.73) or combined with the optional single cell classification an overall accuracy of 86.12(±07.46).

Compared to other methods classifying cancer tissues we achieved improved results. But the direct comparison of the obtained results with previous works is difficult due to different tissue types, preparations methods and staining protocols used in evaluations. Bilgin et al. make use of cell graphs [22] for the classification of chromogenic stained breast cancer tissue samples and reached an overall accuracy of 79.2% on their data set. Breast cancer TMA cores stained with chromogenic dyes where processed in [12] and classification achieved an accuracy of 80% for standard H&E stains and an accuracy of 78% of IHC stained tissues. As the direct comparison of the quantitative results obtained is nearly impossible we here performed the systematic quantitative feature comparison which clearly shows the importance of utilizing all three different feature types. From this we conclude that the challenges arising from working only with DAPI signals in a single color channel can at least well be compensated for by using multiple cell graph feature categories.

The standard deviation of the achieved accuracies in our own data shows an inferior performance on some cores of the SVM. There are several reasons for the performance-loss on such cores. Some cores have a squashed boundary due to the manufacturing of the tissue cores in which cores are pressed into preformed holes. This eventually leads to a higher concentration of cells at border regions which again could lead to a misclassification of these cells due to the wrong topological organization. Another reason for difficulties in processing individual TMAs is that there are stronger intensity alterations between the slides caused by different staining conditions or different imaging conditions such as the exposure time. By standardized experimental protocols this could be avoided in a more routine application in future.

It is expected that the method will require further adaptation of particular parameters and a general improvement when being challenged with tissues of different topological structures. We expect that the performance of our method could decrease on tissues consisting of a greater number of appearingly isolated cells. In such a case the single cell classification based could be improved using for example texture based features. Generally the here presented method could also be deployed for separating not only stroma and tumor but other cell types like lymphocytes. Therefore, the here presented method is an important improvement towards the automated evaluation of new tumor marker in clinical research and diagnostics.
